# Supplementary material for: Analyzing HPV Vaccination Service Preferences among Female University Students in China: A Discrete Choice Experiment
Source: Vaccines (Basel). 2024 Aug 9;12(8):905. doi: 10.3390/vaccines12080905 (PMC11359881; doi:10.3390/vaccines12080905)
Supplement: Supplementary file 1 [file vaccines-12-00905-s001.zip › vaccines-3088542-supplementary.pdf]

Supplementary Materials

Table S1. Preferences of female university students on mixed logit model (N = 1268).

Attributes	Levels	Est	SE
Asc		11.538***	1.206
Effectiveness			
	Normal [c] (ref)		
	Good [d]	1.551***	0.045
	Very good [e]	3.617***	0.081
Protection duration (year)	10 (ref)		
	20	0.936***	0.041
	40	1.547***	0.057
	Lifetime	2.174***	0.072
Waiting time [a] (month)	< 3 (ref)		
	3~6	-0.258***	0.036
	6~9	-0.089**	0.041
	9~12	-0.365***	0.040
Distance [b] (minute)	< 15 (ref)		
	15~30	-0.037	0.037
	30~60	0.116**	0.042
	≥ 60	0.287***	0.038
OOP payment (CNY)		-1.39E-04***	0.000

Note: [a] refers to the waiting time between appointment and successful vaccination of various types of cervical cancer vaccine; [b] refers to the driving time it takes to get to the vaccination site by public transport; [c] prevents cervical cancer only and the prevention effectiveness is about 70%; [d] prevents cervical cancer and genital warts, the prevention effectiveness of cervical cancer is about 70%, genital warts are about 90%; [e] it has preventive effect on cervical cancer and genital warts, and both prevention effectiveness are about 90%; ref: reference; Est: Estimate; SE: Standard error; CNY: Chinese Yuan; OOP: out-of-pocket.

** $p<0.05$; *** $p<0.001$.

Table S2. The AIC and BIC of mixed logit models.

The number of iterations	AIC	BIC
50 draws	18221.27	18450.31
500 draws	18016.44	18245.48
1000 draws	17989.90	18218.94
1500 draws	17983.53	18212.57
2000 draws	17973.67	18202.71
2500 draws	17979.15	18208.19
3000 draws	17982.57	18211.61
3500 draws	17978.54	18207.58
**4000 draws**	**17970.14**	**18199.18**
4500 draws	17975.92	18204.96
5000 draws	17977.66	18206.70

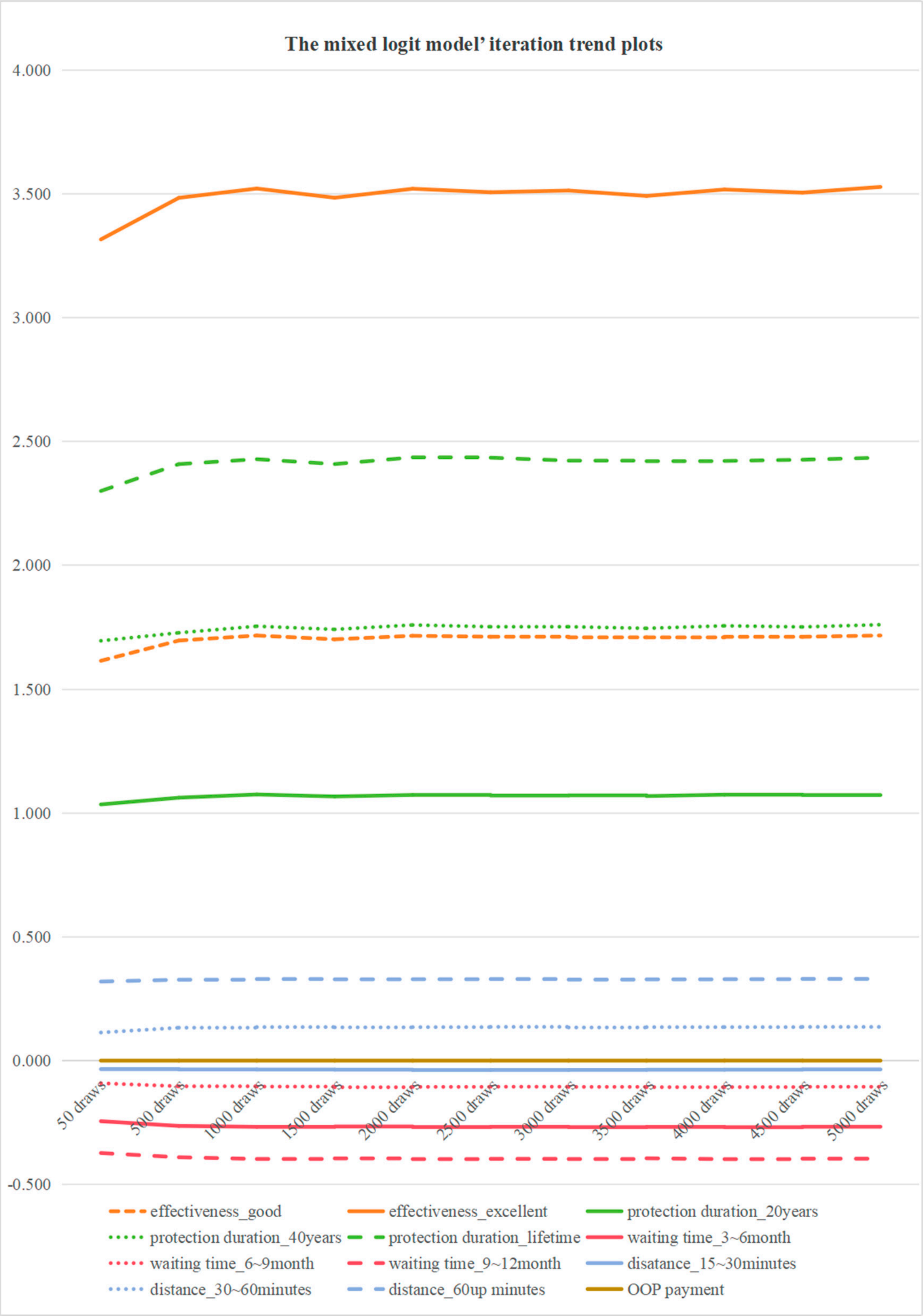

Figure S1. The mixed logit model' iteration trend plots.
